# GPS-MBA: Computational Analysis of MHC Class II Epitopes in Type 1 Diabetes

**DOI:** 10.1371/journal.pone.0033884

**Published:** 2012-03-27

**Authors:** Ruikun Cai, Zexian Liu, Jian Ren, Chuang Ma, Tianshun Gao, Yanhong Zhou, Qing Yang, Yu Xue

**Affiliations:** 1 Hubei Bioinformatics and Molecular Imaging Key Laboratory, Department of Systems Biology, College of Life Science and Technology, Huazhong University of Science and Technology, Wuhan, China; 2 Hefei National Laboratory for Physical Sciences, Microscale and School of Life Sciences, University of Science and Technology of China, Hefei, China; 3 State Key Laboratory of Biocontrol, School of Life Sciences, Sun Yat-sen University, Guangzhou, Guangdong, China; 4 Saban Research Institute of Children's Hospital Los Angeles, Department of Pediatrics, University of Southern California, Los Angeles, California, United States of America; University of Alberta, Canada

## Abstract

As a severe chronic metabolic disease and autoimmune disorder, type 1 diabetes (T1D) affects millions of people world-wide. Recent advances in antigen-based immunotherapy have provided a great opportunity for further treating T1D with a high degree of selectivity. It is reported that MHC class II I-A^g7^ in the non-obese diabetic (NOD) mouse and human HLA-DQ8 are strongly linked to susceptibility to T1D. Thus, the identification of new I-A^g7^ and HLA-DQ8 epitopes would be of great help to further experimental and biomedical manipulation efforts. In this study, a novel GPS-MBA (MHC Binding Analyzer) software package was developed for the prediction of I-A^g7^ and HLA-DQ8 epitopes. Using experimentally identified epitopes as the training data sets, a previously developed GPS (Group-based Prediction System) algorithm was adopted and improved. By extensive evaluation and comparison, the GPS-MBA performance was found to be much better than other tools of this type. With this powerful tool, we predicted a number of potentially new I-A^g7^ and HLA-DQ8 epitopes. Furthermore, we designed a T1D epitope database (TEDB) for all of the experimentally identified and predicted T1D-associated epitopes. Taken together, this computational prediction result and analysis provides a starting point for further experimental considerations, and GPS-MBA is demonstrated to be a useful tool for generating starting information for experimentalists. The GPS-MBA is freely accessible for academic researchers at: http://mba.biocuckoo.org.

## Introduction

Type 1 diabetes (Diabetes mellitus type 1, T1D or T1DM) is a severe chronic autoimmune disease with a relapsing-remitting course that is characterized by the insidious loss of self-tolerance and progressive destruction of insulin-producing pancreatic β-cells in the islets of Langerhans, with the presence of overt hyperglycemia at the time of clinical diagnosis [1]–[7]. The incidence and prevalence of T1D has dramatically increased worldwide over the past several decades, and the onset and development of T1D is believed to be controlled by both genetic and environmental factors [1]–[5], [8]. The cumulative analysis has revealed that a variety of immune cell types, including CD4^+^, CD8^+^ T cells, macrophages and dendritic cells (DCs) are involved in β-cell death, and CD4^+^ T cells play the predominant role in the overall T1D pathology [1], [2], [8]. Thus, the development of immunoregulatory therapeutic approaches has come to be an urgent demand for preventing, treating or even curing T1D [1]–[7].

Besides immunosuppressive drugs and antibody-based immunotherapies, antigen-based “tolerogenic” immunotherapy has attracted considerable attention as a third-generation approach, particularly for its highly selective targeting of aberrant T cells [1]–[6]. It was demonstrated that the MHC class II haplotype, I-A^g7^, is strongly linked to susceptibility to T1D in the non-obese diabetic (NOD) mouse [9]–[11]. Similar linkage to the human HLA-DQ8 molecule, I-A^g7^ is expressed by DCs to present β-cell epitopes from certain well-defined autoantigens, including insulin, glutamic acid decarboxylase (GAD) and insulinoma antigen 2 (IA-2) [1]–[6], [8]. These epitopes are usually composed of 10 to 30 amino acids, with a 9-amino acid core sequence for I-A^g7^/HLA-DQ8 and T-cell receptor (TCR) binding [9]–[11]. In this regard, identification of I-A^g7^/HLA-DQ8 epitopes is fundamental for an understanding of the molecular mechanisms of T1D and the improved design of immunotherapeutic peptides. In 2009, the first-in-human beings Phase I clinical study reported that proinsulin peptide injection is both well tolerated and safe [12]. Recently, a C-peptide deduced from the GAD 65 isoform has generated promising results in Phase II trials, and three Phase III trials are still ongoing [1], [2], [5].

As a complement to labor-intensive and time-consuming experimental assays, the *in silico* prediction of MHC-binding epitopes has emerged as an efficient approach to generate useful information for the purposes of biomedical design [13], [14] (see also http://mba.biocuckoo.org/ links.php). For example, the prediction results of SYFPEITHI [15] and BIMAS [16] were successfully used for the experimental identification of novel MHC class I epitopes derived from type 1 diabetes autoantigens [17]–[19]. Since I-A^g7^ is the only expressed MHC class II molecule in the NOD mouse [9], [10], additional efforts have subsequently been expended on the prediction of I-A^g7^ or HLA-DQ8 epitopes [20]–[23]. In 2006, Rajapakse *et al*. developed the first online server of PRED^NOD^ for the prediction of I-A^g7^, and the two MHC class I molecules K^d^ and D^b^ binding peptides in the NOD mouse [20]. They subsequently refined the predictor using multi-objective evolutionary algorithms (MOEA) [23]. Chang *et al*. used an expectation-maximization alignment algorithm to design computational programs for the prediction of I-A^g7^ [21] and HLA-DQ8 [22] epitopes, respectively. Furthermore, the two integrative tools of MHC2Pred [24], [25] and RANKPEP [26] also include predictors for I-A^g7^ and HLA-DQ8, although they were developed for the comprehensive prediction of a variety of MHC class I and/or II binding peptides. Currently, although a number of computational studies have been performed, only MHC2Pred [24], [25] and RANKPEP [26] are accessible over the internet.

In this work, we developed a novel GPS-MBA software package for the prediction of I-A^g7^ and HLA-DQ8. The experimentally identified epitopes were obtained from the scientific literature, and a modified Gibbs sampling approach was adopted to determine the core nonamers in these epitopes. For training and prediction, a refined GPS algorithm [27], [28] was used. By extensive evaluation and comparison, the prediction performance of GPS-MBA was shown to be highly promising and much better than the other tools currently in use. Moreover, by cross-evaluation using the HLA-DQ8 predictor in GPS-MBA to predict the I-A^g7^ epitopes and *vice versa*, the results show that I-A^g7^ and HLA-DQ8 recognize highly similar peptide profiles. With this powerful tool, we predicted potentially novel I-A^g7^ and HLA-DQ8 binding peptides from T1D-associated epitopes, which bind to other types of MHC molecules. All of the experimentally identified T1D antigens together with their epitopes were absorbed into TEDB 1.0. The *ab initio* predicted epitopes were also provided. Taken together, the prediction and analysis results are helpful for further experimental investigation, and the GPS-MBA can serve as a practically useful adjunct program for experimentalists. The online service and local packages of GPS-MBA 1.0 were implemented in JAVA and freely accessible for academic research purposes at: http://mba.biocuckoo.org.

## Methods

### Data preparation

A search of the scientific literature from PubMed (before Sept. 20^th^, 2011) with the keywords “I-A^g7^ peptide”, “HLA-DQ8 peptide”, or “Type 1 diabetes epitope”, we collected 318 experimentally verified and naturally processed mouse I-A^g7^ binding peptides in 177 proteins, and 134 human HLA-DQ8 epitopes from 84 proteins (Table 1). Additional keywords were tried, but the data set was not changed. The protein sequences were retrieved from the UniProt database (http://www.uniprot.org/uniprot/).

**Table 1:** The statistical data on the experimentally validated epitopes collected in this study.

Experimental data set	Protein	Epitope^a^	Core 9-mer^b^
**Mouse I-A^g7^**	177	318	301
**Human HLA-DQ8**	84	134	127
**Other T1D-associated**	25	203	
**Total**	245	623	

a Epitope, the number of original epitopes;

b Core 9-mer, the number of nonamer core peptides derived from the adapted Gibbs sampling procedure.

In our data set, the length of most of the epitopes varies from 9∼30aa. Thus, we adopted a refined Gibbs sampling approach [29], [30] to determine the 9aa core peptides, and obtained 301 unique nonamer epitopes for the I-A^g7^ and 127 HLA-DQ8 core peptides for training. We also prepared positive (+) and negative (−) data sets for testing. The 318 I-A^g7^ and 134 HLA-DQ8 epitopes of known length were regarded as the (+) set. If at least one predicted nonamer is fully located in the epitope region, the epitope is predicted as a positive hit. To avoid any bias, all of the 9-mer lengths of the same proteins which were either not covered or not fully covered by the original epitopes were taken to be the (−) set.

By further literature review, we collected 203 T1D-associated epitopes in 25 proteins which have the potential to be recognized by other types of MHC or unknown molecules (Table 1).

### Performance evaluation

As previously described [27], [28], we used the four measurements of sensitivity (*Sn*), specificity (*Sp*), accuracy (*Ac*) and Mathew's Correlation Coefficient (*MCC*). The measurements were defined as below:
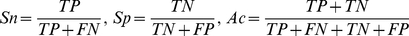
and
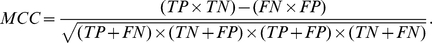



To evaluate the prediction performance and robustness, the leave-one-out (LOO) validation and 4-, 6-, 8- and 10-fold (*n*-fold) cross-validations were performed. In the LOO validation, each core nonamer in the data set was picked out in turn as an independent test sample, and all the remaining core nonamers were regarded as training data. This process was repeated until each nonamer was used as test data one time. In the *n*-fold cross-validation, all the (+) core nonamers and (−) nonamers were mixed and then divided equally into *n* parts, keeping the same distribution of (+) and (−) nonamers in each part. Then *n*-1 parts were merged into a training data set while the remnant part was taken as a testing data set. This process was repeated 20 times and the average performance of *n*-fold cross-validation was computed. Furthermore, the Receiver Operating Characteristic (ROC) curves were drawn, and AROC (area under ROC) values were calculated.

### The algorithm

Previously, we developed a series of GPS algorithms for the prediction of post-translational modification (PTM) sites in proteins [27], [28]. For the prediction of the I-A^g7^ and HLA-DQ8 binding peptides, we used the original method to develop a new algorithm containing two computational parts, a scoring strategy and performance improvement.

The basic hypothesis behind the scoring strategy is that similarly short peptides would exhibit similar 3D structures and biochemical properties [27], [28]. Thus, we can directly use an amino acid substitution matrix, e.g., BLOSUM62, to calculate the similarity between two 9-mer peptides *A* and *B* as:
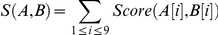



*Score*(*A*[*i*], *B*[*i*]) denotes the substitution score of the two amino acids of *A*[*i*] and *B*[*i*] in the BLOSUM62 at the position *i*. If *S*(*A*, *B*)<0, we redefined it as *S*(*A*, *B*) = 0. A given nonapeptide is then compared with each of the 9aa core peptides from the training data in a pairwise manner to calculate the similarity score. The average value of the substitution scores is regarded as the final score.

The performance improvement procedure is comprised of two steps, weight training (WT) and matrix mutation (MaM). To evaluate the degree of performance improvement, we calculated the scores for all sites of the training data set in each time. By gradually increasing the threshold, we computed the *Sn*, *Sp*, *Ac* and *MCC* under different cut-off values. Thus, we fixed the *Sp* at 90% and compared the *Sn* values of the LOO validation.

#### 1) Weight training (WT)

In this step, the substitution score between the two 9-mer peptides *A* and *B* was updated as follows:
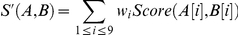



Initially, the weight of each position was defined as 1. The *w_i_* value is the weight of the position *i*. Again, if *S′*(*A*, *B*) is <0, we redefined it as *S′*(*A*, *B*) = 0. Then we randomly picked out the weight of any position for +1 or −1 and re-computed the LOO result. The manipulation was adopted if the *Sn* value was increased. This process was continued until the *Sn* value did not increase any further.

#### 2) Matrix mutation (MaM)

The aim of this step is to generate an optimal or near-optimal scoring matrix from an initial matrix. We re-calculated the LOO result to improve the *Sn* value by randomly picking out an element of the BLOSUM62 matrix for +1 or −1. The process was repeated until convergence was reached. Selecting a different initial matrix, e.g., BLOSUM45 will generate a convergent result if the training time is sufficient (Data not shown).

### Implementation of the online service and local packages

The online service and local packages of GPS-MBA 1.0 were implemented in JAVA. For the online service, we tested GPS-MBA 1.0 on a variety of internet browsers, including Internet Explorer 6.0, 8.0 and 9.0, Mozilla Firefox 8.0 and Google Chrome under the Windows XP and Windows 7 Operating System (OS), Mozilla Firefox 8.0 and Google Chrome under Fedora Core 15 and Ubuntu 10.04 LTS (Linux), and Safari 5.1.1 under Apple Mac OS ×10.5 (Leopard) and 10.7 (Lion). For the Windows and Linux systems, the latest version of Java Runtime Environment (JRE) package (Java SE 5.0 or later versions) should be pre-installed. However, for Mac OS, GPS-MBA 1.0 can be directly used without any additional packages. For convenience, we also developed local packages of GPS-MBA 1.0 which support the three major Operating Systems Windows, Linux and Mac OS X.

## Results

### Determination of the core nonamers from the I-A^g7^ and HLA-DQ8 epitopes

From the scientific literature, we collected 318 naturally processed I-A^g7^ epitopes and 134 HLA-DQ8 binding peptides of various lengths from 8∼30aa (Table 1). The prerequisite for the usage of GPS algorithm is that the length of peptides must be fixed and identical in the training data set [27], [28]. Previously, experimental analyses had suggested that the I-A^g7^ and HLA-DQ8 epitopes contain the 9aa core sequences needed for recognition and binding [9]–[11]. Since there is only one epitope with 8aa, we added one residue upstream and one residue downstream for the 8aa peptide so as to form a decapeptide. Then we used an adapted Gibbs sampling approach to determine the core nonamers of the I-A^g7^ (Table S1) and HLA-DQ8 epitopes (Table S2) [29]–[31].

**Figure 1:**
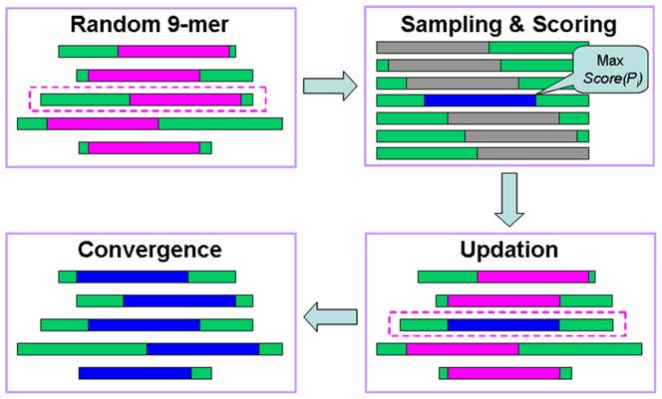
A schematic diagram for the adapted Gibbs sampling approach which was used to determine the nonamer core peptides for I-A^g7^ and HLA-DQ8 epitopes.

Given a set of *N* epitopes *S*_1_, …, *S*_N_, we sought to identify the most probable nonapeptides that were mutually present in both epitopes (Figure 1). First, one 9-mer length per epitope as *P*_1_, …, *P*_N_ was randomly selected, while we randomly singled out one epitope *S*_i_ together with its nonapeptide *P*_i_ (Figure 1). Then we calculated the similarity scores for all of the 9-mer peptides sequentially in *S*_i_, as described below:
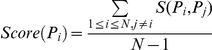



The *Score*(*P*_i_) is the average final similarity score compared with the other nonamers, while *S*(*P*_i_, *P*_j_) is the similarity score between *P*_i_ and *P*_j_. The 9-mer with the maximal *Score*(*P*_i_) is sampled and then updated to the new nonapeptide *P*_i_ (Figure 1). Such a sampling procedure was iteratively repeated until convergence was attained (Figure 1). Ultimately, the redundant 9-mer core peptides were made clearly evident (Table 1, Table S1 and S2).

### Development of GPS-MBA for the prediction of I-A^g7^ and HLA-DQ8 binding peptides

The series of GPS algorithms contain two computational procedures of a scoring strategy and performance improvement [27], [28]. The scoring step has remained the same in all the versions of the GPS algorithms, while the latter process is still in the process of being improved for better performance [27], [28]. In the latest GPS 3.0 release, the performance improvement procedure is comprised of the four sequential steps of *k*-means clustering, motif length selection (MLS), WT and MaM [28]. Originally, we proposed that such a training order could not be changed [28], whereas our recent analysis instead suggests that such an order can be shuffled if the training time is sufficient (Data not shown).

When the training data set is large, the *k*-means clustering approach can be used to classify positive data into multiple groups [28]. However, due to limited data available, this method was not used in this work. The MLS approach was designed for determining the motif length for optimal performance [28]. Since experimental studies suggest that the nonamer core peptides are essential for I-A^g7^ and HLA-DQ8 recognition [9]–[11], this strategy was also not adopted.

**Figure 2:**
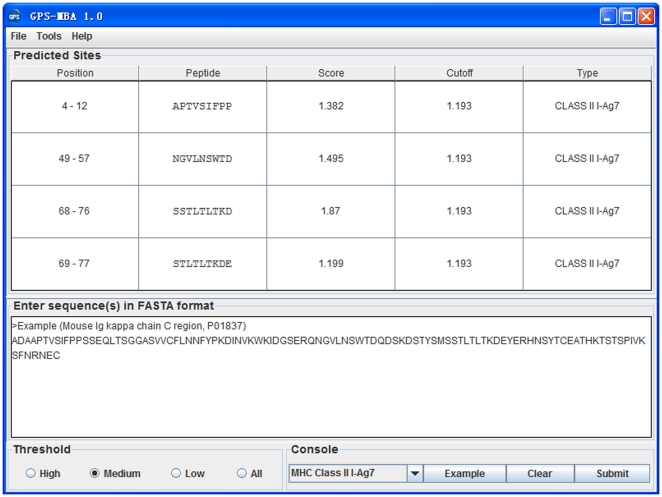
Screen snapshot of the GPS-MBA 1.0 software. The default threshold was chosen for MHC Class II I-A^g7^ (Medium). As an example, the prediction results for the Mouse Igκ chain C region (UniProt ID: P01837) are shown.

Taken together, the GPS algorithm has been improved, and the performance improvement process only required the two steps of WT and MaM. The training order was shuffled for better performance, and the online service and software packages of GPS-MBA 1.0 were implemented in JAVA. As an example, the prediction results for the mouse Igκ chain C region (UniProt ID: P01837) are shown (Figure 2). Previously, a peptide in the mouse Igκ L chain (^174^ERQNGVLNSWTDQDS^188^, identical to 46–60 in the Igκ chain C region) was sequenced as an I-A^g7^ epitope by MS/MS analysis [32]. In our results, four potential I-A^g7^ epitopes of ^4^APTVSIFPP^12^, ^49^NGVLNSWTD^57^, ^68^SSTLTLTKD^76^, and ^69^STLTLTKDE^77^ were predicted (Figure 2). The ^49^NGVLNSWTD^57^ nonamer gives complete coverage of the experimental epitope (46–60), while the other three predicted hits are available for further experimental investigation.

### Performance evaluation and comparison

**Figure 3.The:**
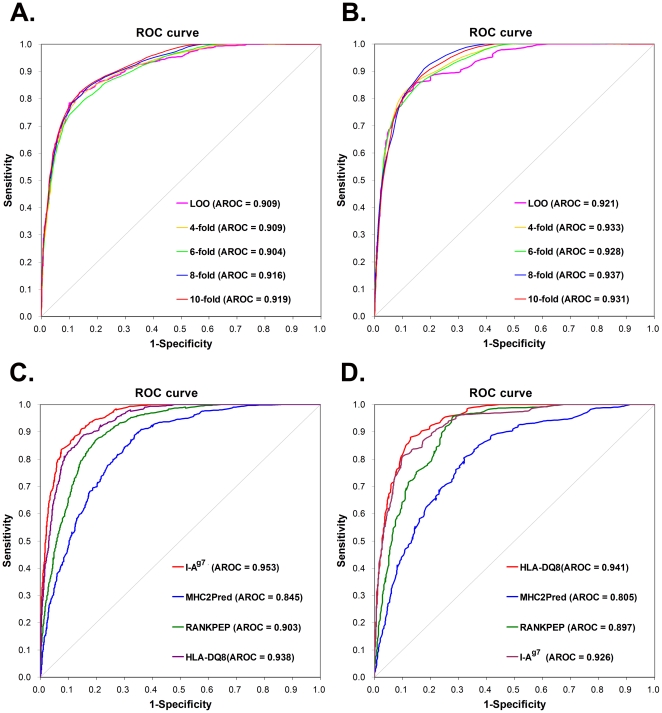
performance evaluation of GPS-MBA 1.0 and a comparison with other approaches. The LOO validation and 4-, 6-, 8- and 10-fold cross-validations were performed for (A) mouse I-A^g7^ and (B) human HLA-DQ8, respectively. We compared the performance of GPS-MBA 1.0 with MHC2Pred and RANKPEP using the LOO validation for (C) I-A^g7^ and (D) HLA-DQ8, respectively. We also performed a cross-evaluation by using the HLA-DQ8 predictor in GPS-MBA to predict (C) I-A^g7^ epitopes and (D) *vice versa*.

To evaluate the prediction performance and robustness of GPS-MBA, LOO validation and 4-, 6-, 8-, 10-fold cross-validations were performed (Figure 3A, 3B). ROC curves were drawn, while the AROC values were 0.909 (LOO), 0.909 (4-fold), 0.904 (6-fold), 0.916 (8-fold) and 0.919 (10-fold) for mouse I-A^g7^ (Figure 3A), and 0.921 (LOO), 0.933 (4-fold), 0.928 (6-fold), 0.937 (8-fold) and 0.931 (10-fold) for human HLA-DQ8 (Figure 3B). Since the results of the 4-, 6-, 8- and 10-fold cross-validations were closely similar to the LOO validation, the GPS-MBA 1.0 is evidently a stable and robust predictor. The performance of the LOO validation was also used for the cut-off setting and further comparison, and the three thresholds of high, medium and low were selected with the *Sp* values of 97%, 95% and 90%, respectively (Table 2). In addition, given the highest *MCC* values, the medium thresholds were chosen as the default thresholds of I-A^g7^ (0.1541) and HLA-DQ8 (0.1534), respectively (Table 2).

**Table 2:** Comparison of GPS-MBA 1.0 with other approaches.

Method	Threshold	Mouse *I-A^g7^*				Human *HLA-DQ8*			
		*Ac* (%)	*Sn* (%)	*Sp* (%)	*MCC*	*Ac* (%)	*Sn* (%)	*Sp* (%)	*MCC*
GPS-MBA	High	96.88	62.89	97.01	0.2066	97.03	51.49	97.17	0.1575
	Medium	94.96	71.70	95.05	0.1829	95.29	64.93	95.38	0.1553
	Low	90.32	84.91	90.34	0.1530	90.11	81.34	90.13	0.1307
Cross^a^		96.83	48.11	97.01	0.1569	97.04	50.00	97.18	0.1532
		94.90	65.09	95.01	0.1646	95.21	60.45	95.32	0.1430
		90.02	82.70	90.05	0.1461	90.09	80.60	90.12	0.1293
MHC2Pred		96.79	23.58	97.07	0.0736	96.90	17.16	97.14	0.0469
		94.79	32.70	95.02	0.0768	94.98	26.87	95.19	0.0564
		89.86	47.80	90.02	0.0763	90.19	44.03	90.33	0.0637
Rankpep		96.77	33.65	97.01	0.1075	96.84	30.60	97.04	0.0886
		94.85	45.91	95.03	0.1130	95.13	40.30	95.30	0.0916
		89.96	64.15	90.06	0.1093	90.02	64.18	90.10	0.0993

For the construction of the GPS-MBA 1.0 software, the three thresholds of high, medium and low were chosen. For comparison, we fixed the *Sp* values of GPS-MBA 1.0 to be identical or similar to other methods and compared the *Sn* values.

a Cross, cross-evaluation by using the HLA-DQ8 predictor to predict I-A^g7^ epitopes and *vice versa*.

To clearly demonstrate the superiority of GPS-MBA, we also used the same data sets to evaluate the performances of MHC2Pred [24], [25] and RANKPEP [26]. We fixed the *Sp* values of MHC2Pred and RANKPEP so as to be similar to GPS-MBA and compared the *Sn* values (Table 2). For I-A^g7^, when the *Sp* value was ∼97%, the *Sn* values of GPS-MBA, MHC2Pred and RANKPEP were 62.89%, 23.58%, and 33.65%, respectively (Table 2). Also, when the *Sp* value was ∼95%, the *Sn* values of GPS-MBA, MHC2Pred and RANKPEP were 71.70%, 32.70%, and 45.91%, respectively (Table 2). Furthermore, when the *Sp* value was ∼90%, the *Sn* of GPS-MBA (84.91%) was still much better than MHC2Pred (47.80%) and RANKPEP (64.15%) (Table 2). Once again, for HLA-DQ8, the GPS-MBA performance is still much better than the other two predictors. In addition, ROC curves were drawn, showing that the AROC values of the GPS-MBA were generally better than the other approaches to I-A^g7^ (Figure 3C) and HLA-DQ8 (Figure 3D).

Previous experimental studies suggested that the mouse I-A^g7^ haplotype is equivalent to the human HLA-DQ8 linkage, and exhibits a similar specificity for peptide recognition and binding [1]–[6], [8]–[11]. To investigate this viewpoint, we performed a cross-evaluation using the HLA-DQ8 predictor in GPS-MBA to predict I-A^g7^ epitopes (Figure 3C) and *vice versa* (Figure 3D). In our results, the cross-evaluation performance is closely similar to the LOO validations (Figure 3C, 3D, and Table 2). Thus, we propose that the binding patterns of I-A^g7^ and HLA-DQ8 are highly similar and conserved.

### Prediction of potentially new I-A^g7^ and HLA-DQ8 epitopes in T1D

Besides I-A^g7^ and HLA-DQ8, certain other MHC class I and II molecules are also implicated in T1D [1]–[7], [17]–[19]. We collected 203 epitopes in 25 proteins from the scientific literature, with 70 MHC class I epitopes, 84 MHC class II binding peptides and 49 epitopes for which the MHC molecules are still undetermined (Table 3, Table S3). Although at present there is a lack of experimental verification, we propose that a number of these epitopes will also come to be recognized by I-A^g7^ and/or HLA-DQ8.

**Table 3:** The statistical data for the prediction of potentially new I-A^g7^ and HLA-DQ8 epitopes in T1D.

Tool	Data set	Protein	Epitope	I-A^g7^		HLA-DQ8		Either	
				Num.^a^	Per.^b^	Num.	Per.	Num.	Per.
**GPS-ARM**	Class I	15	70	1	1.43%	3	4.29%	4	5.71%
	Class II	11	84	31	36.90%	28	33.33%	36	42.86%
	Unknown	5	49	23	46.94%	19	38.78%	28	57.14%
	Total	25	203	55	27.09%	50	24.63%	68	33.50%
**MHC2Pred**	Class I	15	70	5	7.14%	2	2.86%	7	10.00%
	Class II	11	84	25	29.76%	24	28.57%	42	50.00%
	Unknown	5	49	22	44.90%	14	28.57%	29	59.18%
	Total	31	203	52	25.62%	40	19.70%	78	38.42%
**RANKPEP**	Class I	15	70	14	20.00%	3	4.29%	15	21.43%
	Class II	11	84	42	50.00%	24	28.57%	48	57.14%
	Unknown	5	49	28	57.14%	18	36.73%	33	67.35%
	Total	31	203	84	41.38%	45	22.17%	96	47.29%

a Num., the number of manually collected epitopes predicted with core 9-mers.

b Per., percentiles.

To predict potential I-A^g7^ and HLA-DQ8 epitopes, we used GPS-MBA 1.0 with the default thresholds. In total, 68 epitopes were predicted to interact with either I-A^g7^ or HLA-DQ8 (33.5%) (Table 3). Also, the prediction results of MHC2Pred [24], [25] and RANKPEP [26] were present with default thresholds (Table 3). In particular, the prediction performance of GPS-MBA 1.0 is much poorer in terms of the MHC class I epitopes, such that only one and three epitopes were predicted as I-A^g7^ and HLA-DQ8 binding peptides, respectively (Table 3). However, GPS-MBA 1.0 displayed a considerably effective performance for MHC class II epitopes by predicting 31 I-A^g7^ and 28 HLA-DQ8 binding peptides (Table 3). Also, the distributions of results from MHC2Pred and RANKPEP are similar (Table 3). In this regard, the sequence profiles of MHC class I and II are quite different, whereas GPS-MBA, MHC2Pred and RANKPEP have the capacity to efficiently distinguish both MHC class I and II epitopes. In particular, GPS-MBA, MHC2Pred and RANKPEP predicted 28 (57.14%), 29 (59.18%) and 33 (67.35%) unannotated epitopes as having either I-A^g7^ or HLA-DQ8 binding peptides (Table 3). Taken together, this analysis suggests that there are additional *bona fide* I-A^g7^ and HLA-DQ8 epitopes which still remain to be identified, and these prediction results comprise a useful resource for further experimental investigation. The detailed prediction results of GPS-MBA are shown in Table S1.

### The development and usage of TEDB 1.0

To provide an integrative platform for computational analysis of the T1D epitopes, all of the experimental identified T1D-associated antigens together with their epitopes were collected for the development of TEDB (Table 4). Also, we used GPS-MBA 1.0 at the default threshold to predict potential I-A^g7^ or HLA-DQ8 epitopes in 245 antigens (Table 4). In addition, the prediction results of MHC2Pred and RANKPEP were also included in the TEDB 1.0.

**Table 4:** The statistical results of the TEDB 1.0 database.

Organism	Protein	Known epitopes	Predicted epitopes			Total
			I-A^g7^	HLA-DQ8	Either	
***H. Sapiens***	20	262	553	527	773	944
***M. musculus***	180	285	4,627	4,701	6,587	6,485
**R. norvegicus**	13	18	331	382	505	503
**Others**	32	58	801	785	1,123	1,105
**Total**	245	623	6,312	6,395	8,988	9,037

**Figure 4:**
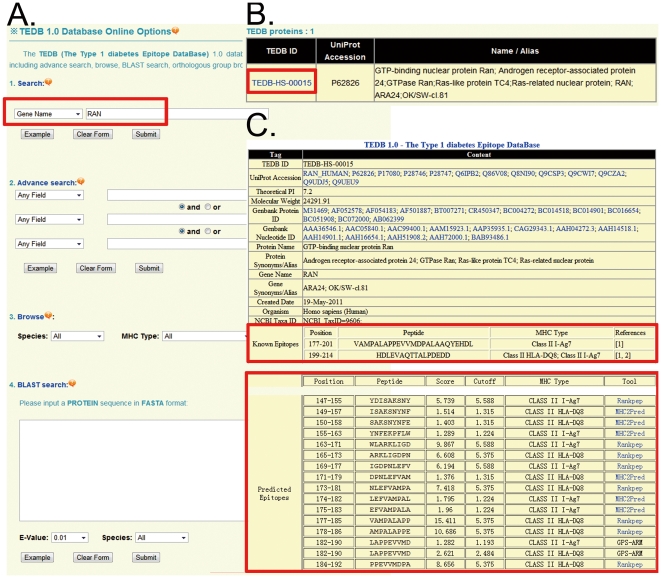
The search options for the TEDB 1.0 database. (A) Users are able to simply input ‘RAN’ and select “Gene Name” for querying. (B) The results are shown in a tabular format. Users can then click on the TEDB ID (TEDB-HS-00015) to visualize the detailed information. (C) The detailed information on human RAN. The experimentally identified and predicted epitopes are presented.

TEDB 1.0 was developed in a user-friendly manner. The search option (http://mba.biocuckoo.org/database.php) provides an interface for querying the TEDB database with one or several keywords such as TEDB ID, MHC Type, or UniProt Accession, etc (Figure 4A). We also provided three advance options, including advance search, browse and BLAST search (Figure 4A). For example, if the keyword ‘RAN’ is inputted and submitted (Figure 4A), the result is shown in a tabular format, with the features of TEDB ID, UniProt accession, and protein/gene names/aliases (Figure 4B). By clicking on the TEDB ID (TEDB-HS-00015), the detailed information on human RAN is shown (Figure 4C). The experimentally identified epitopes and predicted I-A^g7^ and HLA-DQ8 binding peptides are provided, while the protein sequence, Gene Ontology annotation, domain organization, molecular weight and computed/theoretical *I*_p_ are also provided (Figure 4C).

## Discussion

T1D, also termed Insulin-dependent diabetes mellitus (IDDM) or juvenile diabetes, is a chronic metabolic disorder and autoimmune disease that takes a heavy toll world-wide [1]–[7], [33]. Currently, it is estimated that approximately 70,000 children develop T1D per year, ∼30,000 of which cases are in the United States, and an annual global increase of ∼3% [4], [5], [33]. Although a number of immunotherapeutic and regenerative approaches have been developed, the effective prevention and treatment of T1D are both still a great challenge [1]–[7], [33]. Recent advances in the development of antigen-based “tolerogenic” immunotherapy have provided a great opportunity for treating T1D with a high degree of selectivity, while two investigations are still ongoing as clinical trials, and the outcome is uncertain [1]–[7], [12], [33]. In this regard, the Identification of T1D-speicific epitopes is needed for further experimental and biomedical design.

Although a number of computational analyses were carried out for the prediction of the I-A^g7^ or HLA-DQ8 epitopes, the online services have been not available over the internet [20]–[23]. Again, although the two integrative tools of MHC2Pred and RANKPEP do contain predictors of I-A^g7^ and HLA-DQ8, they were actually designed for the general prediction of MHC class I and/or II epitopes [24]–[26]. Thus, they might exhibit a sensitive performance for other MHC molecules, and yet not I-A^g7^ and/or HLA-DQ8. In this study, we focused on the prediction of I-A^g7^ and HLA-DQ8 by constructing the GPS-MBA software package. By comparison, the performance of GPS-MBA is much better than MHC2Pred and RANKPEP (Table 2, Figure 3C, 3D).

Originally, the series of GPS algorithms were developed for the prediction of PTM sites in proteins [27], [28], and this is the first use of the algorithm for MHC epitopes. The major difference between PTM sites and MHC epitopes is that the PTM site positions are anchored by the middle residues, while the lengths and positions of the MHC epitopes are promiscuous and difficult to fix. In this regard, the MHC epitope core sequences of defined length must be determined prior to training. Originally, the Gibbs sampling method was designed for detecting short conserved motifs from multiple DNA or protein sequences [29]–[31]. By utilizing the position-specific scoring matrices (PSSMs), the amino acids frequencies were counted in the foreground and background data sets, respectively. In this procedure, the ratio of the pattern probability to the background probability was calculated and improved by sampling, until convergence was attained [29]–[31]. However, the average similarity score, but not the frequency ratio, was calculated for further sampling in our analysis. The 9-mer core peptides were determined by this approach for I-A^g7^ and HLA-DQ8, respectively.

**Figure 5:**
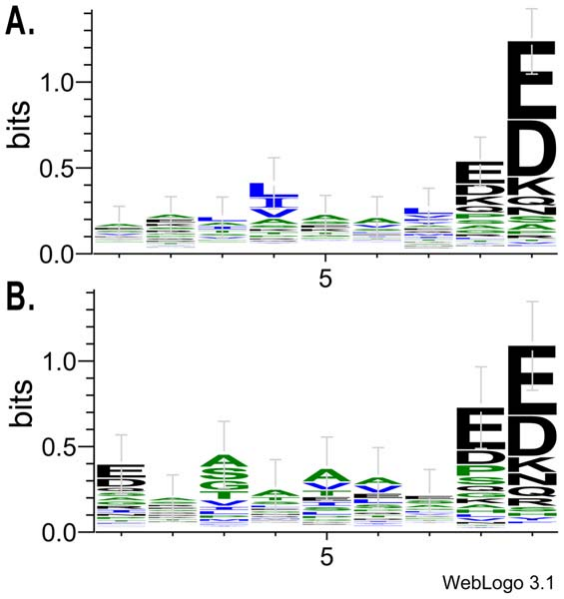
The sequence logos of the core nonamers for (A) I-A^g7^ and (B) HLA-DQ8.

Here, we used the WebLogo server [34] to analyze the sequence profiles of the core nonamers for I-A^g7^ (Figure 5A) and HLA-DQ8 (Figure 5B), respectively. Previously, experimental studies based on limited epitopes had suggested that P4, P6 and P9 are three conserved positions, while P1 is a degenerate position [9]–[11]. However, our results suggest that P9 is the most informative position, along with a less the comparatively weakly conserved position of P8 (Figure 5A, 5B). P4 is weakly conserved in I-A^g7^ core nonamers (Figure 5A) and not conserved in HLA-DQ8 (Figure 5B). Although the sequence logo of I-A^g7^ is not evidently similar to HLA-DQ8, our cross-evaluation results indicate that I-A^g7^ and HLA-DQ8 are able to recognize similar peptide profiles (Table 2, Figure 3C, 3D). We speculated that whether most of the HLA-DQ8 epitopes would also be I-A^g7^ binding peptides in the training data set, since the NOD mouse is the best model for T1D. However, there are only 24 epitopes in the original data set which interact with both I-A^g7^ (24/318) and HLA-DQ8 (24/134). Again, in the non-redundant core nonamers, only 22 9-mer peptides were found to bind with both I-A^g7^ and HLA-DQ8. Based on these results, we propose that the sequence profile of I-A^g7^ is intrinsically similar to that of HLA-DQ8.

In addition to the predictions of GPS-MBA, all of the experimentally validated T1D-associated epitopes were collected in the online database of TEDB 1.0. The *ab initio* predictions with GPS-MBA were also integrated into TEDB. Thus, such an integrative platform should prove to be useful for experimentalists. We believe that computational analysis, together with subsequent experimental identification, will help advance the study of T1D into a new and highly productive phase.

## Supporting Information

Table S1 **The core nonamers of the mouse I-A^g7^ epitopes.** *a*. Epitope, the original epitopes; *b*. Position, the positions of core nonamers in the protein sequences; *c*. Core nonamer, the finally aligned core nonamers were marked in red; *d*. The protein sequence was retrieved from the UniParc (UniProt Archive) Database (http://www.uniprot.org/help/uniparc).
(XLS)

Table S2 **The core nonamers of the human HLA-DQ8 epitopes.**
(XLS)

Table S3 **From the scientific literature, we collected 203 epitopes in 25 proteins, with 70 MHC class I epitopes, 84 MHC class II binding peptides, and 49 epitopes for which the MHC molecules are still unknown.** The detailed prediction results of GPS-ARM were provided. *a*. UniProt, the UniProt accession numbers of T1D antigens; *b*. Pos., the position of the original known epitopes; *c*. Peptide, the experimentally identified epitopes; *d*. MHC Type, the experimentally identified MHC molecules that recognize the epitopes; *e*. Pre. Pos., the predicted position of the binding peptides; *f*. Pre. Peptide, the predicted core nonamers; *g*. Pre. Type, the predicted MHC molecules of I-A^g7^ and HLA-DQ8 that potentially recognize the core 9-mers.
(XLS)
