# Correction: GPS-MBA: Computational Analysis of MHC Class II Epitopes in Type 1 Diabetes

**DOI:** 10.1371/annotation/97a13c7b-1037-4293-bf15-be18d0550f0c

**Published:** 2012-08-09

**Authors:** Ruikun Cai, Zexian Liu, Jian Ren, Chuang Ma, Tianshun Gao, Yanhong Zhou, Qing Yang, Yu Xue

The funding statement should read: This work was supported by grants from the National Basic Research Program (973 project) (2010CB945400, 2012CB911200,2012CB910101), Natural Science Foundation of China (90919001, 31071154, 30900835, 30830036, 91019020, 31171263, 30971642), Natural Science Foundation of Hubei Province (2009CDA161), and Fundamental Research Funds for the Central Universities (HUST: 2010JC049, 2010ZD018, 2011TS085; SYSU: 11lgzd11). The funders had no role in study design, data collection and analysis, decision to publish, or preparation of the manuscript. 

